# Delta Radiomics Analysis for Local Control Prediction in Pancreatic Cancer Patients Treated Using Magnetic Resonance Guided Radiotherapy

**DOI:** 10.3390/diagnostics11010072

**Published:** 2021-01-05

**Authors:** Davide Cusumano, Luca Boldrini, Poonam Yadav, Calogero Casà, Sangjune Laurence Lee, Angela Romano, Antonio Piras, Giuditta Chiloiro, Lorenzo Placidi, Francesco Catucci, Claudio Votta, Gian Carlo Mattiucci, Luca Indovina, Maria Antonietta Gambacorta, Michael Bassetti, Vincenzo Valentini

**Affiliations:** 1 Fondazione Policlinico Universitario “Agostino Gemelli” IRCCS, 00168 Rome, Italy; davide.cusumano@policlinicogemelli.it (D.C.); luca.boldrini@policlinicogemelli.it (L.B.); angelaromano89@gmail.com (A.R.); antoniopiras88@gmail.com (A.P.); giuditta.chiloiro@policlinicogemelli.it (G.C.); lorenzo.placidi@policlinicogemelli.it (L.P.); francesco.catucci@guest.policlinicogemelli.it (F.C.); claudio.vt@hotmail.it (C.V.); giancarlo.mattiucci@policlinicogemelli.it (G.C.M.); luca.indovina@policlinicogemelli.it (L.I.); mariaantonietta.gambacorta@policlinicogemelli.it (M.A.G.); vincenzo.valentini@policlinicogemelli.it (V.V.); 2 Department of Human Oncology, School of Medicine and Public Health, University of Wisconsin-Madison, 600 Highland Ave, Madison, WI 53792, USA; yadav@humonc.wisc.edu (P.Y.); bassetti@humonc.wisc.edu (M.B.); 3 Department of Oncology, University of Calgary, 1331 29 Street NW, Calgary, AB T2N 1N4, Canada; sangjune.lee@ucalgary.ca

**Keywords:** radiomics, pancreatic cancer, magnetic resonance, MR-guided radiotherapy

## Abstract

The aim of this study is to investigate the role of Delta Radiomics analysis in the prediction of one-year local control (1yLC) in patients affected by locally advanced pancreatic cancer (LAPC) and treated using Magnetic Resonance guided Radiotherapy (MRgRT). A total of 35 patients from two institutions were enrolled: A 0.35 Tesla T2*/T1 MR image was acquired for each case during simulation and on each treatment fraction. Physical dose was converted in biologically effective dose (BED) to compensate for different radiotherapy schemes. Delta Radiomics analysis was performed considering the gross tumour volume (GTV) delineated on MR images acquired at BED of 20, 40, and 60 Gy. The performance of the delta features in predicting 1yLC was investigated in terms of Wilcoxon Mann–Whitney test and area under receiver operating characteristic (ROC) curve (AUC). The most significant feature in predicting 1yLC was the variation of cluster shade calculated at BED = 40 Gy, with a *p*-value of 0.005 and an AUC of 0.78 (0.61–0.94). Delta Radiomics analysis on low-field MR images might play a promising role in 1yLC prediction for LAPC patients: further studies including an external validation dataset and a larger cohort of patients are recommended to confirm the validity of this preliminary experience.

## 1. Introduction

Despite the progress in diagnosis and the development of multimodal therapies, pancreatic cancer still represents the fourth leading cause of cancer death in the world, with more than 80% of patients presenting unresectable or metastatic disease at diagnosis and a 5-year overall survival rate of 10–20% [1,2].

For patients affected by locally advanced pancreatic cancer (LAPC), the first therapeutic approach is to date represented by combined systemic chemotherapy [3,4,5]. Treatment intensification with locoregional multimodal therapies such as high-intensity focused ultrasound (HIFU), chemoradiotherapy (CRT), or stereotactic body radiation therapy (SBRT) in patients showing stable disease or considered not resectable after chemotherapy induction is still a controversial issue in the oncological community [6,7,8].

The advantages of subsequent CRT in terms of local control (LC) and overall survival (OS) were firstly suggested by retrospective studies but not confirmed in perspective trials, leaving open the question on its real clinical impact [9,10,11,12].

In the framework of radiotherapy, the recent introduction of advanced on-board imaging systems that are able to ensure the delivery of higher doses to the tumour without compromising the functionality of the radiosensitive organs at risk (OARs) nearby has led to an increased role of SBRT in the clinical management of LAPC patients, making this strategy a valuable option in the multimodal treatments [13,14,15,16,17,18,19].

In this context, the recent introduction of magnetic resonance guided radiotherapy (MRgRT) opened a new frontier, offering the possibility to deliver daily online adaptive treatments, able to effectively compensate inter-fraction variation of the tumour and surrounding OARs. In addition, the on-board MR imaging available on the hybrid units ensures better anatomical definition of therapy volumes, thanks to the higher soft tissue contrast and continuous tumour motion monitoring during treatment delivery by means of a planar MR acquisition in cine modality [20,21].

In particular, the possibility of online adapting the treatment plan appears to be of crucial importance in the clinical management of LAPC patients to ensure the most appropriate treatment every day, taking into consideration inter-fraction and intra-fraction tumour variability [22,23,24].

In the framework of the modern ‘omics’, radiomics is playing a relevant role, identifying image-based biomarkers that are able to predict clinical outcomes and showing significant results since the first years of its application in different treatment sites, such as the pelvis, abdomen, and thorax [25,26,27,28,29].

In particular, a variant of this approach, called Delta Radiomics, studying the variation of the radiomics parameters during treatment, seems to be able to model the patient treatment response during therapy, reporting promising results in diseases as gastrointestinal and pancreatic cancers [30,31].

The aim of this study was to investigate the role of Delta Radiomics in predicting the one-year local control (1yLC) in patients affected by LAPC and treated using MRgRT, identifying new image-based biomarkers obtained by the quantitative analysis of the on-board MR images acquired during treatment.

## 2. Materials and Methods

### 2.1. Patients

Patients affected by LAPC who underwent MRgRT using a low-field MR-linear accelerator (MRIdian, ViewRay, Mountain View, CA, USA) in two different institutions were retrospectively enrolled.

Specific informed consent to MRgRT treatment and Magnetic Resonance Imaging (MRI) safety screening forms were administered to all patients before therapy. Patients younger than 18 years, with the presence of metastasis at diagnosis, with clinical contraindication to MRI (e.g., presence of non-MRI-safe implanted devices, claustrophobia, affected by psychiatric disorders) or denying specific consents, were excluded from this study.

Patients enrolled in centre A (Wisconsin University, Madison, WI, USA) received four different dose schedules: 35 Gy, 40 Gy, or 50 Gy in 5 fractions and 67.5 Gy in 15 fractions, based on the clinical situation of the patient at the time of diagnosis and on the distance between the target and OARs at the simulation.

All the patients enrolled in centre B (Fondazione Policlinico Universitario Agostino Gemelli IRCCS, Rome, Italy) received 40 Gy in 5 fractions [24,32].

All patients underwent induction chemotherapy regimens that included Gemcitabine, Gemcitabine plus INN-Paclitaxel, FOLFIRINOX (Oxaliplatin at the first day; Irinotecan at the first day; Folinic Acid, at the first and second day; 5-Fluorouracil at the first day; and 5-Fluorouracil at the first and second day in continuous infusion; each 14 days), and Capecitabine regimens.

For the MRgRT treatment, a true fast imaging with steady state precession (TrueFISP) MR sequence was acquired each day of therapy, using a sequence of 25 s in breath hold inspiration and resulting in MR images characterised by a T2*/T1 weighted image contrast, with a field of view (FOV) of 54 × 47 × 43 cm^3^, an in-plane spatial resolution of 1.5 × 1.5 mm^2^, and a slice thickness of 3 mm [33]. The MR acquisition protocol adopted was identical in both the institutions.

### 2.2. Image Analysis

Gross tumour volume (GTV) and organs at risks were manually delineated during simulation and propagated on each daily MR image acquired for patient positioning: based on the daily anatomy, the clinician decided whether to adapt the Radiotherapy (RT) treatment plan or deliver the original one calculated on the MR image acquired during simulation.

Delta Radiomics analysis was focused on GTV as the region of interest (ROI) and one-year local control (1yLC) as outcome. In particular, 1yLC was defined in case that the patient one year after the end of the MRgRT treatment showed a GTV volume less or equal to the one measured at the simulation.

To adequately compare the radiomics features extracted from MR images acquired at different times, physical dose values were converted to biologically effective dose (BED) assuming an alpha/beta value of 10 Gy [34].

The quantitative analysis considered the GTV delineated on the MR images acquired at the BED levels of 20, 40, and 60 Gy: based on the fractionation scheme chosen for the MRgRT treatment, the fractions reported in Table 1 were selected for the analysis.

**Figure 1:**
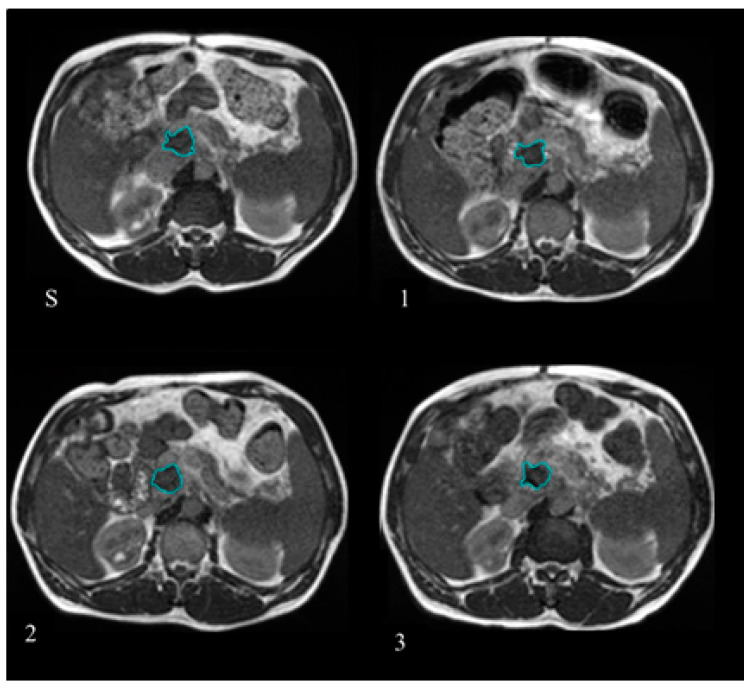
Gross tumour volume (GTV) delineated at the treatment simulation (**S**) and at the different treatment fractions selected for the Delta Radiomics analysis, corresponding to BED levels of 20 Gy (**1**), 40 Gy (**2**), and 60 Gy (**3**). The GTV contours are reported in blue lines

GTV delineation was retrospectively checked for anatomical consistency on the MR images selected for the analysis using the MIM software (version 6.7.6 MIM Software Inc., Cleveland, OH, USA) by two radiation oncologist experts in the gastrointestinal malignancies. Figure 1 reports the example of one patient where the GTV was delineated at simulation and at the treatment fractions selected to have BED levels of 20, 40, and 60 Gy.

The DICOM files containing the MR images and the RT structure file were exported and processed using MODDICOM, an R package developed for radiomic analysis [35,36]. A total of 92 radiomics features were extracted from each single MR image, without applying any kind of image filter or pre-processing on the raw images. The extracted radiomic features belonged to three families: Intensity-based (19), morphological-based (13), and textural-based (60).

As regards the textural features, 25 of them are based on grey-level co-occurrence matrix (GLCM), 15 of them are based on grey-level run length matrix (GLRLM), and 16 are based on the grey-level size zone (GLDZM). All the image features analysed met the standardisation criteria of the IBSI initiative: the complete list of the features is reported in Supplementary Materials [37].

In addition to the original radiomic features extracted by the single MR images, the variation of each radiomic feature with respect the corresponding value reported in simulation was calculated and considered as delta radiomic feature.

The Wilcoxon Mann–Whitney (WMW) test was used to investigate the ability of these features in predicting 1yLC at univariate analysis, and the feature showing the lowest *p*-value was considered as the most predictive parameter.

Receiver operating characteristic (ROC) curve analysis was performed on the most significant feature obtained at the WMW analysis, calculating the area under curve (AUC) with the corresponding 95% confidence interval (CI) following the Clopper–Pearson method [38].

In absence of an external validation dataset, the robustness of the delta radiomics feature identified was evaluated by means of a five folds cross-validation analysis with tree iterations [39].

In order to provide a general overview of the clinical cohort under investigation, the correlation between five clinical parameters (age, clinical staging following TNM classification (cT, cN, cM), and total BED) and 1yLC was also reported using the WMW test. Such analysis was repeated considering also the overall survival one year after the end of treatment (1yOS) as outcome.

## 3. Results

A total of 35 patients were enrolled in this retrospective hypothesis-generating study: 28 patients from Institution A and seven patients from Institution B.

All patients were diagnosed with pancreatic adenocarcinoma, except one who had a ductal pancreatic adenocarcinoma. Seven patients showed the presence of metastases at diagnosis: two of them were oligometastatic, one had two metastases, and the others had three or more metastases. Metastases were mainly located in liver (71%), with one patient having a single metastasis in the lung and one with two metastases in the liver and peritoneum.

On the whole cohort of patients, a total of 18 patients (51.4%) were treated with 67.5 Gy in 15 fractions, 9 cases (25.7%) were treated with 35 Gy in 5 fractions, 7 cases (20%) were treated with 40 Gy in 5 fractions, and one case was treated with 50 Gy in 5 fractions (2.8%).

A total of 18 patients received an adaptive treatment, while the others received a conventional SBRT treatment without daily adaptation. Clinical characteristics of the patients enrolled are reported in Table 2.

Out of a total of 35 patients analysed, 24 of them were alive one year after the end of treatment (1yOS = 69%), while 20 of them had local control (1yLC = 57%).

A total of 644 features (368 radiomic and 276 delta features) were calculated for each patient: the univariate analysis performed using the WMW test identified seven features showing a *p*-value less than 0.05 in predicting 1yLC. The most significant feature of 1yLC analysis was the variation of the cluster shade calculated on the co-occurrence matrix when a BED value of 40 Gy is reached, reporting a *p*-value of 0.005.

**Figure 2:**
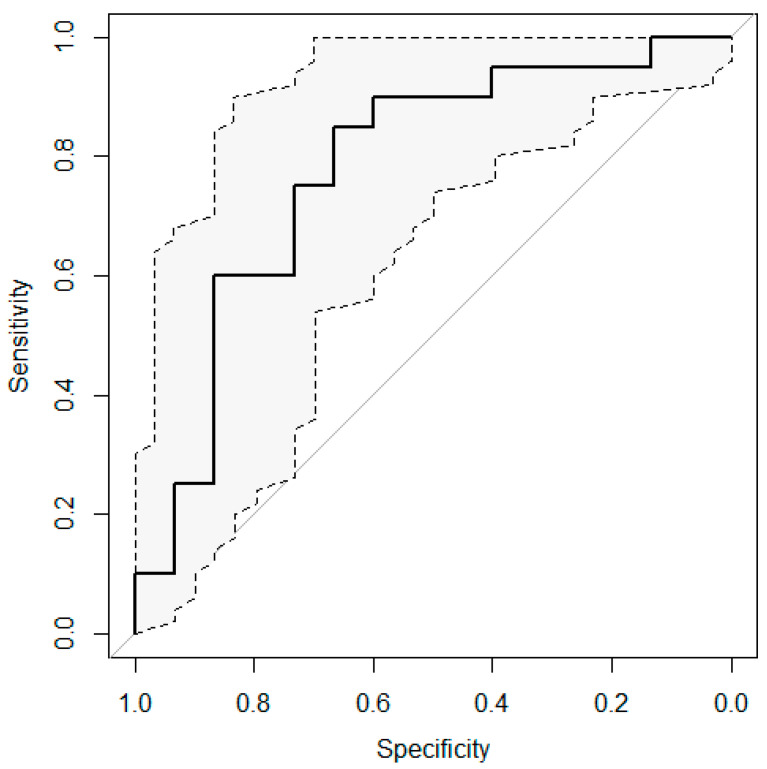
Receiver operating characteristic (ROC) curve calculated for the variation of the cluster shade feature when a BED value of 40 Gy is reached The ROC curve is reported in continue line, the extremes of the 95% confidence interval are reported in dot lines.

The ROC curve obtained considering the discriminative performance of this delta feature is shown in Figure 2: an AUC value of 0.78 was obtained, with a 95% confidence interval ranging from 0.61 to 0.94.

The cross-validation analysis reported an AUC of 0.79 with a confidence interval ranging from 0.62 to 0.97. As regards the analysis of the clinical variables, no significant correlation was observed considering 1yLC and 1yOS as outcome: the detailed results in terms of *p*-values obtained considering the WMW test are reported in Table 3.

## 4. Discussion

Outcome prediction through radiomics analysis of on-board MR images is a topic of great interest in the scientific community due to the very promising clinical results achieved in the pancreatic cancer care using MRgRT [40,41].

Models able to provide reliable predictions during the RT course can be of great value to clinicians, allowing timely change of the dose prescription, moving towards dose escalation, or alternative approaches for patients indicated by the model as not-responders, in the perspective of response-based treatment adaptation [42].

The first Delta Radiomics experiences in the pancreatic cancer were reported by Nasief et al., who developed on 90 patients a machine learning process that was able to early predict the response to CRT treatment by analysing the temporal variation of radiomic features extracted by CT images acquired every day during the therapy [31].

Then, the same authors combined the previously described Delta Radiomic model with a widely used clinical biomarker for pancreatic cancer (CA19-9), demonstrating how the integration of the two predictors leads to more reliable predictive performance than considering the individual parameters alone [43].

As regards the use of MR imaging for treatment response prediction, the main experiences were focussed on the radiomic analysis of MR images acquired at a single time point: Kaissis et al. proposed a model able to predict the overall survival by analysing the pre-operative diffusion weighted images, while Tang et al. elaborated a model for early recurrence prediction from the analysis of pre-operative T2 and T1-weighted MR images [44,45,46].

As regards the Delta Radiomics MR-based models, the only experience to the best of our knowledge is represented by the study of Simpson et al., who showed how the delta approach on 0.35 T MR images may have a promising role in tumour response prediction of pancreatic cancer patients [47].

On a cohort of 20 patients, Simpson et al. have elaborated a machine learning method able to identify in advance the patients who will have a complete response to MRgRT treatment: despite the similar endpoints with respect our analysis, substantial differences can be recognised.

First of all, in our experience, a larger number of patients was analysed (35), and the identification of a single feature was preferred to the elaboration of a machine learning model, with the aim of obtaining a single image-based biomarker that could have a clinical and biophysical interpretation, reducing the risk of model overfitting. Secondly, the BED approach was adopted to compensate for different radiotherapy schemes, thus extending the generalisability of the proposed approach to different dose prescriptions [48,49].

The most significant feature emerged in our analysis was the variation of the cluster shade when a BED value of 40 Gy was reached, which is an indicator of a textural variation occurred inside the GTV during the course of MRgRT: further studies including known biomarkers (such as lab test or markers levels etc.) are needed to determine which biological substrate this clinical evidence is related to. Such feature showed an AUC of 0.78 (95% CI: 0.61–0.94) on the cohort analysed, which was confirmed by the cross-validation analysis (AUC = 0.79, 95% CI: 0.62–0.97): the reason of the large CI widths observed are mainly due to the low numerosity of the sample analysed.

However, the experience reported in this study represents a hypothesis-generating study, as it was conducted on a limited cohort of patients and does not include an external validation dataset, which represents a mandatory step to demonstrate the generalisability of this approach and moving towards the clinical application: the lack of an external dataset was partially compensated by means of a cross-validation approach, which confirmed the high discriminative performance of the identified feature.

The whole image analysis was performed on the raw MR images acquired for patient positioning, without applying any image pre-processing operations: the impact of image filters will be investigated in future studies, as several MR-based and CT-based radiomic experiences have demonstrated their utility in the enhancement of feature extraction [50,51,52].

Another limitation of this experience is represented by the lack of an analysis related to the reproducibility of the radiomic features, which represents a necessary step for the clinical implementation in studies based on manual segmentation processes. In this context, a great evolution is expected from automatic contouring systems that are able to ensure precise and standardised contours in a very short time: the feasibility of these approaches on MRI has been recently demonstrated by some experiences, even focused on the abdominal district [53,54].

Finally, it is noteworthy that no statistical significance emerged in the analysis between the clinical variables considered and one-year survival and local control: this could be due to the short time considered for the outcomes observation and to the low numerosity of the sample analysed.

Despite the limited number of patients considered, hypothesis-generating studies are proving to be able to obtain interesting findings, which often represent the starting point for more structured research. An interest example in this framework is represented by the experience of Gill et al., who recently observed in 15 melanoma patients a significant correlation between the radiomic features extracted by CT images and the concentration of circulating tumour DNA in blood plasma through a dedicated statistical analysis ad hoc developed for the purpose of the study [55].

The experience described in this paper has to be considered within the context of these pilot studies, with findings that confirm the potentialities of low-field MR imaging for Delta Radiomics applications, encouraging to set up a MRgRT consortium to further explore the predictive role of Delta Radiomics analysis in treatment personalisation using daily MR acquisitions.

## 5. Conclusions

Delta Radiomics analysis performed on low-field MR images appeared to be a feasible and promising image analysis technique for the determination of 1yLC probability in patients affected by pancreatic cancer. Further studies including a larger cohort of patients, automatic segmentation procedures, and an external validation set are recommended to confirm the preliminary results here reported.

Considering the ability of Delta Radiomics approaches to represent the patient’s treatment response through the variation of the clinical images acquired on a daily basis, the use of these systems is expected to grow in the future, providing physicians with increasingly reliable systems to support their clinical decisions and paving the way towards more precise and personalised cancer approaches.

## Figures and Tables

**Table 1:** Fractions selected to obtain equivalent biologically effective dose (BED) levels to varying of the prescription dose and fractions.

Prescription Dose and Fractions (fx)	BED = 20 Gy	BED = 40 Gy	BED = 60 Gy	Total BED
35 Gy in 5 fx	Fraction 2 (BED = 23.8 Gy)	Fraction 3 (BED = 35.7 Gy)	Fraction 5 (BED = 59.5 Gy)	59.5 Gy
40 Gy in 5 fx	Fraction 1 (BED = 14.4 Gy)	Fraction 3 (BED = 43.2 Gy)	Fraction 4 (BED = 57.6 Gy)	72 Gy
50 Gy in 5 fx	Fraction 1 (BED = 20 Gy)	Fraction 2 (BED = 40 Gy)	Fraction 3 (BED = 60 Gy)	100 Gy
67.5 Gy in 15 fx	Fraction 3 (BED = 19.6 Gy)	Fraction 6 (BED = 39.2 Gy)	Fraction 9 (BED = 58.7 Gy)	97.9 Gy

**Table 2:** Patient characteristics and outcomes distribution for the patients included in the study. 1yLC: Local control after 1 year since the end of the treatment.

	Number of Patients
Age (range)	69 (55–88)
Gender	
Female	14 (40%)
Male	21 (60%)
Histology	
Adenocarcinoma	34 (97%)
Ductal Adenocarcinoma	1 (3%)
Clinical Tumour Staging (T)	
cT1	2 (6%)
cT2	12 (34%)
cT3	8 (23%)
cT4	13 (37%)
Clinical Nodal Staging (N)	
cNx	2 (6%)
cN0	20 (57%)
cN1	9 (25%)
cN2	2 (6%)
cN3	2 (6%)
Clinical Metastasis Staging (M)	
cM0	28 (80%)
cM1	7 (20%)
Structures Involved by Tumour at Diagnosis	
None	2 (6%)
Duodenum	1 (3%)
Pancreas	11 (30%)
Liver	2 (6%)
Vessels	13 (37%)
Pancreas and Vessels	2 (6%)
Duodenum and Vessels	2 (6%)
Duodenum, Stomach and Vessels	1 (3%)
Pancreas, Duodenum and Stomach	1 (3%)
1yLC	20 (57%)
1yOS	24 (69%)

**Table 3:** Results of the univariate analysis performed using the Wilcoxon Mann–Whitney (WMW) test between five clinical variables (cT, cN, cM, age, and BED) and two outcomes (one-year local control, 1yLC; and one-year overall survival, 1yOS).

Clinical Variable	Univariate Analysis (*p*-Value)	
	1yLC	1yOS
Clinical Tumor Staging (cT)	0.82	0.74
Clinical Nodes Staging (cN)	0.68	0.57
Clinical Metastasis (cM)	0.10	0.29
Age	0.17	0.28
Biologically Effective Dose (BED)	0.76	0.91

## Data Availability

The raw data supporting the conclusions of this article will bemade available by the authors, without undue reservation.

## Supplementary Materials

The Supplementary Materials are available online at https://www.mdpi.com/2075-4418/11/1/72/s1.
