# Supplementary material for: Delta Radiomics Analysis for Local Control Prediction in Pancreatic Cancer Patients Treated Using Magnetic Resonance Guided Radiotherapy
Source: Diagnostics (Basel). 2021 Jan 5;11(1):72. doi: 10.3390/diagnostics11010072 (PMC7824764; doi:10.3390/diagnostics11010072)
Supplement: Supplementary file 1 [file diagnostics-11-00072-s001.pdf]

Supplementary materials

The complete list of the 92 features extracted is reported below, divided by family. The id reported in the list is the same reported in the biomarker initiative refence manual, where it is possible to found the mathematical formulation of each feature [36].

- **Morphological-based**
    - 4.1.1 Volume
    - 4.1.3 Surface area
    - 4.1.4 Surface to volume ratio
    - 4.1.5 Compactness 1
    - 4.1.6 Compactness 2
    - 4.1.7 Spherical disproportion
    - 4.1.8 Sphericity
    - 4.1.9 Asphericity
    - 4.1.10 Centre of mass shift
    - 4.1.11 Maximum 3D diameter
    - 4.1.12 Major axis length
    - 4.1.13 Minor axis length
    - 4.1.14 Least axis length

- **Intensity-based**
    - 4.1.15 Elongation
    - 4.1.16 Flatness
    - 4.3.1 Mean
    - 4.3.2 Variance
    - 4.3.3 Skewness
    - 4.3.4 Kurtosis
    - 4.3.5 Median
    - 4.3.6 Minimum grey level
    - 4.3.7 10th percentile
    - 4
    - .3.8 90th percentile
    - 4.3.9 Maximum grey level
    - 4.3.10 Interquartile range
    - 4.3.11 Range
    - 4.3.12 Mean absolute deviation
    - 4.3.13 Robust mean absolute deviation
    - 4.3.17 Energy
    - 4.3.18 Root mean square
    - 4.4.18 Entropy
    - 4.4.19 Uniformity

- **Grey level co-occurrence based features–Texture features (GLCM)**
    - 4.6.1 Joint maximum
    - 4.6.2 Joint average
    - 4.6.3 Joint variance
    - 4.6.4 Joint entropy
    - 4.6.5 Difference average
    - 4.6.6 Difference variance
    - 4.6.7 Difference entropy
    - 4.6.8 Sum average
    - 4.6.9 Sum variance
    - 4.6.10 Sum entropy
    - 4.6.11 Angular second moment
    - 4.6.12 Contrast
    - 4.6.13 Dissimilarity
    - 4.6.14 Inverse difference
    - 4.6.15 Inverse difference normalised
    - 4.6.16 Inverse difference moment
    - 4.6.17 Inverse difference moment normalised
    - 4.6.18 Inverse variance
    - 4.6.19 Correlation
    - 4.6.20 Autocorrelation
    - 4.6.21 Cluster tendency
    - 4.6.22 Cluster shade
    - 4.6.23 Cluster prominence
    - 4.6.24 First measure of information correlation
    - 4.6.25 Second measure of information correlation

- **Grey level run length based features–Texture features (GLRLM)**
    - 4.7.1 Short runs emphasis
    - 4.7.2 Long runs emphasis
    - 4.7.3 Low grey level run emphasis
    - 4.7.4 High grey level run emphasis
    - 4.7.5 Short run low grey level emphasis
    - 4.7.6 Short run high grey level emphasis
    - 4.7.7 Long run low grey level emphasis
    - 4.7.8 Long run high grey level emphasis
    - 4.7.9 Grey level non-uniformity
    - 4.7.10 Grey level non-uniformity normalised
    - 4.7.11 Run length non-uniformity
    - 4.7.12 Run length non-uniformity normalised
    - 4.7.13 Run percentage

- 4.7.14 Grey level variance
- 4.7.15 Run length variance
- 4.7.16 Run entropy

- **Grey level size zone based features–Texture features (GLDZM)**
    - 4.8.1 Small zone emphasis
    - 4.8.2 Large zone emphasis
    - 4.8.3 Low grey level zone emphasis
    - 4.8.4 High grey level zone emphasis
    - 4.8.5 Small zone low grey level emphasis
    - 4.8.6 Small zone high grey level emphasis
    - 4.8.7 Large zone low grey level emphasis
    - 4.8.8 Large zone high grey level emphasis
    - 4.8.9 Grey level non-uniformity
    - 4.8.10 Grey level non-uniformity normalised
    - 4.8.11 Zone size non-uniformity
    - 4.8.12 Zone size non-uniformity normalised
    - 4.8.13 Zone percentage
    - 4.8.14 Grey level variance
    - 4.8.15 Zone size variance
    - 4.8.16 Zone size entropy
